# *De-novo* Domestication for Improving Salt Tolerance in Crops

**DOI:** 10.3389/fpls.2021.681367

**Published:** 2021-09-16

**Authors:** Ali Razzaq, Fozia Saleem, Shabir Hussain Wani, Shaimaa A. M. Abdelmohsen, Haifa A. Alyousef, Ashraf M. M. Abdelbacki, Fatemah H. Alkallas, Nissren Tamam, Hosam O. Elansary

**Affiliations:** ^1^Centre of Agricultural Biochemistry and Biotechnology, University of Agriculture, Faisalabad, Pakistan; ^2^Division of Genetics and Plant Breeding, Sher-E-Kashmir University of Agricultural Sciences and Technology of Kashmir, Srinagar, India; ^3^Physics Department, Faculty of Science, Princess Nourah bint Abdulrahman University, Riyadh, Saudi Arabia; ^4^Applied Studies and Community Service College, King Saud University, Riyadh, Saudi Arabia; ^5^Plant Production Department, College of Food and Agriculture Sciences, King Saud University, Riyadh, Saudi Arabia

**Keywords:** climate change, *de novo* domestication, salinity tolerance, crop wild relatives, genome editing, pan-genomes, next-generation sequencing, halophytes

## Abstract

Global agriculture production is under serious threat from rapidly increasing population and adverse climate changes. Food security is currently a huge challenge to feed 10 billion people by 2050. Crop domestication through conventional approaches is not good enough to meet the food demands and unable to fast-track the crop yields. Also, intensive breeding and rigorous selection of superior traits causes genetic erosion and eliminates stress-responsive genes, which makes crops more prone to abiotic stresses. Salt stress is one of the most prevailing abiotic stresses that poses severe damages to crop yield around the globe. Recent innovations in state-of-the-art genomics and transcriptomics technologies have paved the way to develop salinity tolerant crops. *De novo* domestication is one of the promising strategies to produce superior new crop genotypes through exploiting the genetic diversity of crop wild relatives (CWRs). Next-generation sequencing (NGS) technologies open new avenues to identifying the unique salt-tolerant genes from the CWRs. It has also led to the assembly of highly annotated crop pan-genomes to snapshot the full landscape of genetic diversity and recapture the huge gene repertoire of a species. The identification of novel genes alongside the emergence of cutting-edge genome editing tools for targeted manipulation renders *de novo* domestication a way forward for developing salt-tolerance crops. However, some risk associated with gene-edited crops causes hurdles for its adoption worldwide. Halophytes-led breeding for salinity tolerance provides an alternative strategy to identify extremely salt tolerant varieties that can be used to develop new crops to mitigate salinity stress.

## Climate Changes and Crop Domestication

Food security remains the top priority for all the stakeholders and policymakers to ensure food availability to everyone, while climate changes and rapid population growth are the major obstacles to achieve this goal (Lesk et al., 2016; Myers et al., 2017). It is a huge challenge to feed the continuously booming population, which is expected to reach 10 billion and need 49% more food at the end of 2050 (Ray et al., 2013). Currently, 820 million people are facing extreme hunger, and this number could increase abruptly in the coming years and can worsen the global status of food security (FAOSTAT, 2020). The numbers of undernourished people have also increased in the last 6 years both in relative and absolute terms. The hidden hunger in Western Asia, Africa, and other underdeveloped countries is much higher now as compared to a decade ago (FAO, 2018).

In addition, unprecedented weather conditions linked with environmental degradation and increased land competitions due to urbanization are hampering the crop production (Lobell and Gourdji, 2012; Lenaerts et al., 2019). Climate change is anticipated to increase the temperature of earth that causes global warming, uneven rain spells, and intensifies several abiotic and biotic stresses which are drastically reducing the crop yield (Raza et al., 2019). It is predicted that the climate changes will be more prevalent and intensify different stresses in the future and pose serious threats to agricultural production. So, it will be very hard to sustain the current rate of crop yields (Ray et al., 2013). The annual crop yield must be accelerated in order to keep the sustainable crop production despite the challenges of climate stresses and population growth (Tilman et al., 2011; Ray et al., 2013). These multi-dimensional factors need a major transformation of agricultural productions systems (Lenaerts et al., 2019). The utilization of sustainable resources to increase the crop yield per unit area and use of water and fertilizer more efficiently can assist for sustainable crop production.

Availability of enough food largely relies on economic growth crucial to lessen the hidden and chronic hunger; however, it might not be adequate to eliminate the hunger (Gödecke et al., 2018). Plant breeding has been one fundamental strategy to meet the food demands of people through crop domestication for thousands of years (McKersie, 2015). Crop domestication is an evolutionary ongoing process based on rigorous screening and selection of best-performing cultivars to improve agronomic traits and better acclimatization by growing the cultivated, landraces crop wild relatives (CWRs) (DeHaan et al., 2020). It helps to develop new crop species or modify the domesticated crop species that can withstand different environments and give high crop yields (Purugganan, 2019). Also, domestication can modify the genetic makeup of different domesticated varieties at a genomic level and transform them into climate-resilient species (Dawson et al., 2019). Numerous agronomic traits have been introduced into several populations through domestication process in the pre-genetic era (Lavarenne et al., 2018). The green revolution in the 1960s led to an remarkable boost in crop output per area and put greater pressure for sustainable agriculture production (Østerberg et al., 2017). These conventional breeding approaches are unable to keep the pace for crop yield in post-green revolution due to reduction in genetic diversity, and crops became more susceptible to climatic stresses (Tilman et al., 2002). Also, there are several disadvantages associated with traditional breeding approaches such as genetic erosion, genetic drag, hybridization incompatibilities, and time consuming screening processes, as it requires 15–20 years to develop a new crop variety (Fischer et al., 2014; Abberton et al., 2016).

The development of climate-smart varieties that have the potential to withstand diverse environmental condition is inevitable to tackle these climate changes and ensure food security. Development of genetically modified (GM) crops through transgenic technology is one of the modern breeding strategies that can cross all the barriers faced by conventional breeding approaches and help to mitigate develop climate resilient crops (Raman, 2017). There are numerous examples of GM crops that has been commercialized in the United States after the development of the first GM crop “flavor savor tomato” in 1994 (James, 1997). To date, more than 500 events in 33 GM crops have been reported and commercialized by the Food and Drug Administration (FDA) in different countries, with the USA as the leading country to grow GM crops. The area under GM crops cultivation has significantly increased in the last decade and 91% of total GM area is shared among five countries including USA, Canada, Argentina, Brazil, and India (ISAAA Database, 2021). Almost all of the traits introduced in GM crops are related to herbicide resistant, insect resistant, and very little reports on drought and salinity stress tolerance which are multi-genic in nature. Apart from this, GM crops are banned by European and some other countries and there are several concerns linked with this technology which include possible negative effects on non-targeted organisms, increased resistance in insect/pathogens, human biosafety, and commercialization related problems (Paul et al., 2018). So, keeping in view the stated concerns, transgenic technology is imperfect and we need alternative fast-forward molecular breeding approaches to enhance genetic gains in crop domestication and develop climate-resilient crops (Marsh et al., 2021; Razzaq et al., 2021a).

In this review, we discuss the impact of salinity tolerance on crop production with a major focus on salinity tolerance mechanism. We highlight the importance of CWRs due to their greater genetic pool and spotlight some salt tolerant genes harbor by CWRs. We describe the advance techniques like pangenomes and transcriptome profiling to study the salt tolerant mechanism and identification of stress-responsive genes in CWRs. We argue that *de novo* domestication is a promising strategy that should be employed to develop salt tolerance varieties using genome editing tools. We elaborate the significance of halophytes that can provide an alternative route for breeding salinity tolerant crops. We also discuss the pitfall remains and propose future outlooks.

## Salinity Stress Tolerance

Among several abiotic stresses, salt stress is a major limiting factor that greatly threatens crop production globally, turning into a huge concern for plant breeders (Wani et al., 2020). As climate changes are predicted to increase in the coming years, soil salinity can become more severe because of varied irrigation practices and lead to the deterioration of heavily irrigated arable soils. Also, soil salinization is the main problem for areas having inadequate water supply or limited irrigation facilities (Akram et al., 2017). More than 1,125 million hectares of total arable area are affected by salinization in 13 countries including Australia, China, India, USA, Israel, Mexico, Turkey, Egypt, Bangladesh, Pakistan, Syria, Iran, and Iraq (Hossain, 2019).

High salinization can drastically impede crop growth and decrease the root's potential to uptake water and nutrients from the soil and cause ion cytotoxicity and osmotic stress (Yang et al., 2020). Salinization is also induced by oxidative stress because of the generation of reactive oxygen species (ROS). The induction of salt stress can be triggered by two phases: ionic toxicity and osmotic stress. The later one is a quick action because of the elevation in osmotic pressure, while the ion toxicity is the result of hyper-accumulation of Na^+^ in cytosol responsible for sodium (Na^+^) and chloride (Cl^−^) imbalance (Hanin et al., 2016). Both phases are assumed to be separated spatially and temporally, with Na^+^ reducing water uptake while slow accumulation of Na^+^ inhibits photosynthesis activity in the shoots (Van Zelm et al., 2020).

Salinity stress can bring undesirable modifications in chemical properties of cell wall *via* modulating the gene expression indirectly or Na^+^ directly interacting with different components of cell wall (Munns and Gilliham, 2015). The higher level of Na^+^ in apoplast can enhance the chances of physical interaction among positive and negative charges and control pH of apoplast (Farooq et al., 2017). This ion imbalance causes cellular dysfunction, variation in membrane integrity, ion exclusion, metabolic syndrome, nutritional ailments, poor seed germination, injuries to young seedlings, reduce plant vigor, cell senescence, and stunted plant growth (Morton et al., 2019).

**Figure 1:**
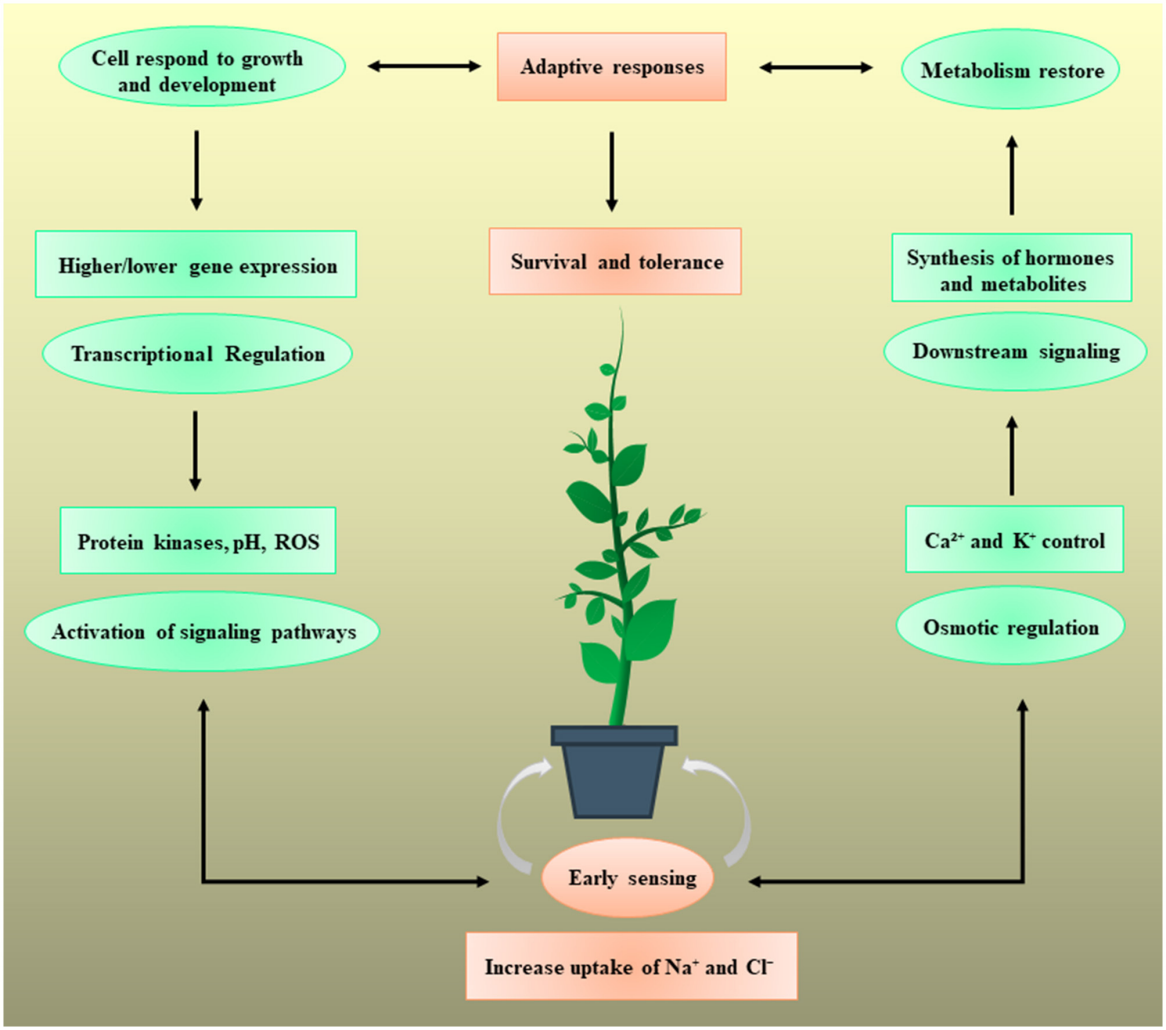
Plants respond to salt stress by triggering different regulatory mechanisms and assist the plants for their survival. The Na^+^ and Cl^−^ bring particular downstream responses, but the plant sensing systems are not yet fully understood. The mechanism could start with the early sensing of Na^+^ import intracellularly or extracellularly. This early sensing induces the different osmotic and regulatory pathways including protein kinase activity, reactive oxygen species (ROS), pH balance, H^+^ transport, and Ca^2+^ and K^+^ signaling. These early sensing mechanisms reduce the Na^+^ import and initiate the downstream signaling pathways to activate the transcriptional machinery which may increase or decrease the expression of certain stress-responsive genes. It also causes significant difference in the concentration, synthesis, and transport of unique hormones and metabolites. Finally, the adaptive response aids the plant cells to bypass the salinity stress by modulation of growth and metabolism. Each step of salt inducive mechanism plays a vital role in providing the salinity tolerance and survival to the plant.

**Figure 2:**
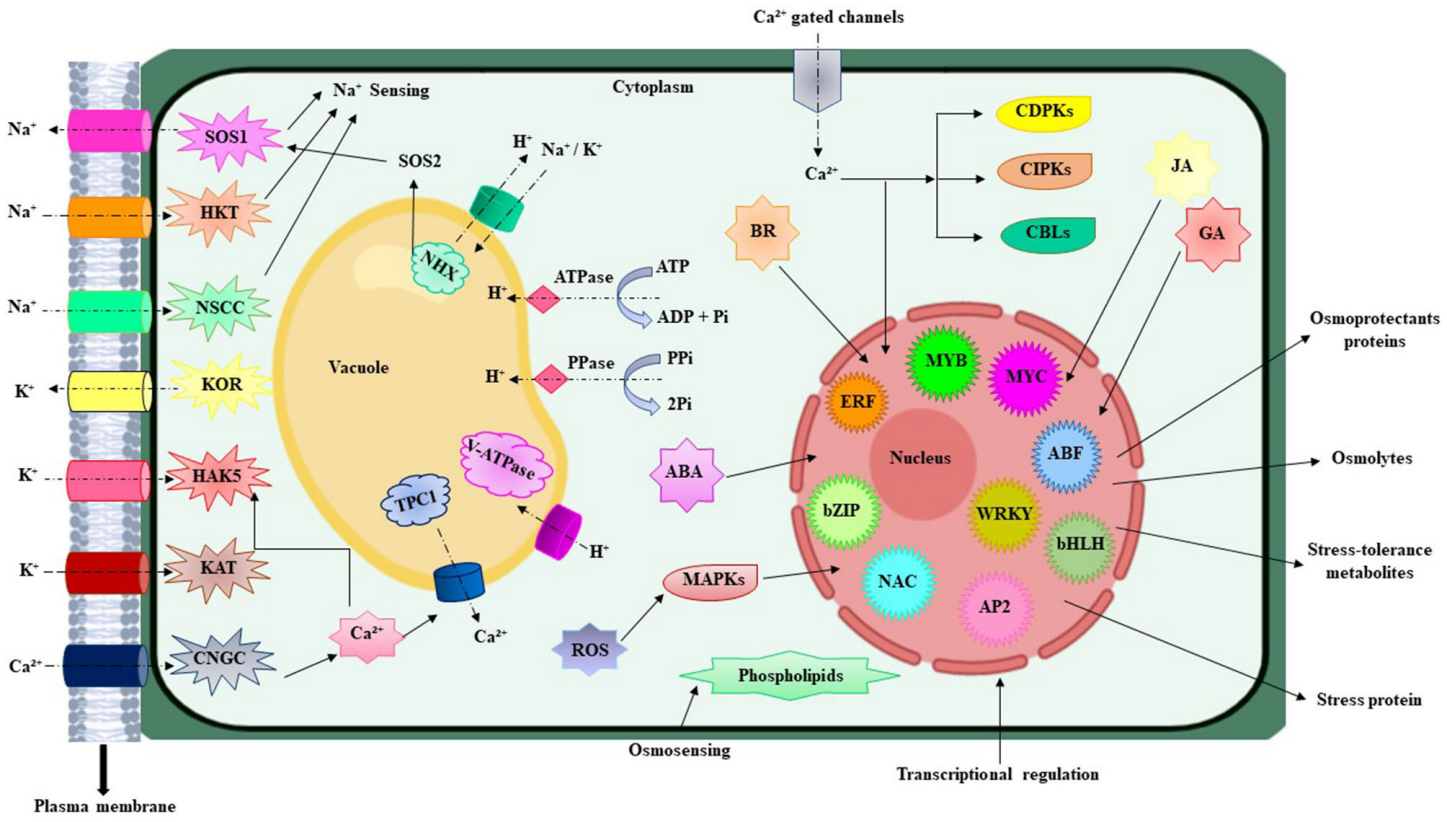
Illustrated the simplified overview of different component for salt response and salt tolerance mechanism in plants. Na^+^ cross the plasma membrane through CNGC, HKT, NSCCs, and some other unknown gated channels (showed as dotted black arrows). This ion imbalance causes the modification in membrane voltage flux and disturbs the inflow and outflow of Ca^2+^ and K^+^ ion. After the Na^+^ import into the cell, different unidentified ionic and hyperosmotic sensory networks initiate the early sensing. This resulted in the activation of certain Ca^2+^ gate channels and triggered the detoxification pathways *via* HKT, KOR, KAT, HAKS, and antiporter SOS1 and maintains the ion balance within the cell. SOS1 moves out the Na^+^ from the root cortex while HKTI allows the reverse flux and discharge Na^+^ from xylem to stop the hyperaccumulation in photosynthetic cells. The ionic stress is also tackled *via* sequestration by means of NHX channels that import the K^+^ or Na^+^ and moves out the H^+^ from the vacuole. H^+^ ions are also transported by the V-ATPase, PPase, and ATPase in opposite direction. Ca^2+^ export from the vacuole through TPC1 and helps to maintain the ionic and osmotic balance. At the next step, Ca^2+^ produces ROS that showed a positive correlation between them. This activated the signaling pathways of CDPKs, CIPKs, and CBLs which after entry imitates the transcriptional modification. The expression of several gene families is controlled at transcriptional level including MYB, MYC, ERF, bZIP, NAC, WRKY, ABF, AP2, and bHLH change which ultimately provide salt stress tolerance to plants. Also, ABA-mediated pathways triggered the production of JA, BR, and GA that helps the plant to recover from the salinity syndrome. NSCCs, non-selective cation channels; SOS, salt overly sensitive; HKT, high-affinity potassium transporter; KOR, potassium outward-rectifying; HAKs, high-affinity K+ transporters; CNGC, cyclic nucleotide-gated ion channel; NHX, sodium hydrogen exchanger; TPC1, two-pore Channel 1; ROS, reactive oxygen species; MAPKS, mitogen activated protein kinases; CIPKs, CBL-interacting protein kinases; CBLs, calcineurin Blike proteins; CDPKs, calcium-dependent protein kinases; bZIP, basic leucine zipper; AP2/ERF, Apetala2/ethylene response factor; JA, jasmonic acid; BR, Brassinosteroids; GA, Gibberellic acid. Signs: Ion transport (dotted arrow), promotion (arrow).

During evolution, multidimensional counter mechanisms for achieving salinity tolerance have been adapted by the plants for survival. A general mechanism of plant stress tolerance is depicted in Figure 1. Salt-tolerant mechanism starts with the early sensing of Na^+^ which induces downstream mechanisms to initiate counter processes to deal with the salinity stress. A salt tolerance mechanism is comprehensively described and illustrated in Figure 2. Soil salinity negatively influences root development and plants have generally developed two key strategies to cope with the salinization based on either restricting the entry of NaCl into the roots or by diluting the salt concentration in the cell through increase water uptake (Hanin et al., 2016). At the cellular and molecular level roots regulate the gene expression, metabolism, and protein expression, which modulate the cell wall functions, transport mechanisms, stabilize roots shape and size to deal with the unfavorable environment (Byrt et al., 2018). Plants have also acquired specialized mechanisms for sensing the ionic and hyperosmotic conditions to manage the salinity stress, but the molecular bases behind these sensing properties are yet to be explored (Wani et al., 2020).

Thus, the major purpose is to elucidate target traits responsible for overall salinity stress tolerance. For this we need to understand the functional expression of key genes *via* holistic approaches which allow salinity tolerance to break down into fragments that are genetically chaseable (Demidchik and Maathuis, 2007; Deolu-Ajayi et al., 2019). Better knowledge about the flexibility of salinity tolerance mechanism across a wide range of environmental conditions is crucial for further dissection. The need for advanced breeding approaches is being aided by the parallel progress in next-generation sequencing (NGS) and high-throughput phenotyping tools that are now allowing comprehensive assessment of huge genetic databanks (Morton et al., 2019). Numerous genes and transcription factors (TFs) are controlling the salinity tolerance mechanism alongside with complex regulatory metabolic pathways. Many TF gene families have been discovered that govern salt tolerance and some TFs are supposed to regulate several abiotic stresses, particularly salinization (Dai et al., 2018). Exploring the hidden cellular and molecular networks and their corresponding genes controlling the salt accumulation and transportation will open exciting avenues to develop salt tolerant varieties to meet food security challenges in the prevailing trends of climate change (Genc et al., 2016). This can only be achieved by rapid gene identification and recapturing the unique genes from wild relatives and halophytes that have been lost during the domestication process by using advanced molecular breeding approaches (Liu et al., 2020a).

## *De novo* Domestication

With the advent of advanced technologies, *de novo* domestication has been considered as a viable and alternate strategy for crop improvement, while sustaining the agricultural yield in limited resources for ensuring food security (Hoyos et al., 2020). In many crop species, CWRs adapted to stress conditions give appropriate genetic stock for *de novo* domestication (Zsögön et al., 2018). *De novo* domestication can be described as the incorporation of domesticated genes into the non-domesticated species to develop new crops. Hence, instead of incorporating domesticated genes from CRWs into cultivated species, as in conventional approaches, *de novo* domestication is done to mimic the natural process of breeding (Fernie and Yan, 2019). *De-novo* domestication strategy may offer quick and accurate modifications of CWRs into cultivated crops, while preserving most of the beneficial alleles controlling stress resilience, important agronomic and nutritional traits, disappeared during breeding (Gasparini et al., 2021; Razzaq et al., 2021b).

*De novo* domestication can be vital to overcome the certain genetic obstacles, including improvement in the facilities of diversified natural ecosystem, build strong relationship among specific ecological niches and crops, helping the crops to withstand under harsh or extremely stressful climatic conditions, and increase the genetic diversity and genetic gains of agricultural systems (Shelef et al., 2017). The information acquired from investigating the domestication process in model species can be crucial to study the CWRs and helps to obtain inclusive knowledge about the genomes (Kang et al., 2016). Domestication for monogenic traits is comparatively easy to carry out, but abiotic stress tolerance traits are polygenic in nature and very tough to harness (Stitzer and Ross-Ibarra, 2018). For example, in the case of salt tolerance, it contains several pathways of regulating the crop growth like Na+ inclusion and exclusion process in the cell to source-sink connection of whole plant. So, the CWRs containing stress-tolerant traits can be *de novo* domesticated *via* targeting the monogenic genes by applying cutting-edge genome editing toolbox. Beneficial polygenic genes expressing in CWRs having stress resistance are dispersed and hard to mutate (Khan et al., 2019).

The idea of *de novo* domestication is to explore and examine the stress-responsive genes by mutating them that resulted in the designing of new crop varieties having better adaptation and climate resilience (Razzaq et al., 2021b). Genomes of important crop species have been sequenced and some are in the pipeline that also includes underutilized crops, CWRs halophytes, and models plants (Jackson et al., 2011; Kang et al., 2016). With the availability of more sequenced genomes, it becomes obvious to unravel the genetic architecture and exploit these data sets to identify the domestication genes for *de novo* domestication.

**Figure 3:**
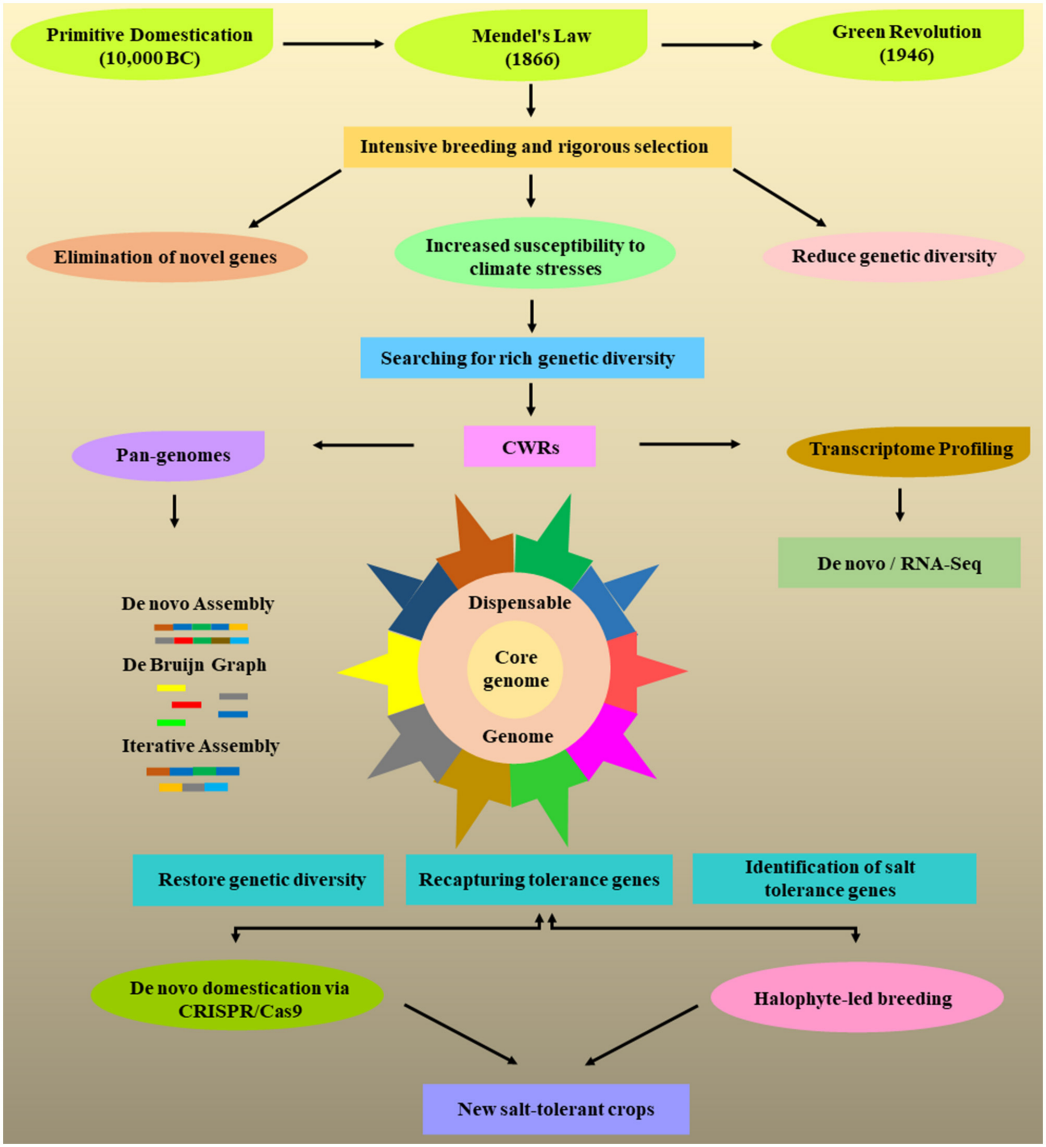
Portrayed some major events in the history of plant breeding to increase the crop production. Like primitive domestication leads to the discovery of Mendel's law to study the plant genetics and green revolution that select the specific agronomic traits to increase the crop yield. This intensive domestication led to the reduction in genetic diversity and crops become more susceptible to abiotic and biotic stresses due to the elimination of resistant genes. The crop wild relatives (CWRs) are vital to decipher the stress-responsive genes and can be exploited for transcriptional profiling of several unique salt tolerance genes through next-generation sequencing (NGS) technologies. The landraces cultivated and CWRs are used to construct the high quality pangenomes for crop improvement. The pangenomes consist of core and dispensable genomes representing all the genes present within a species. It helps to increase the genetic diversity of a species and recapture the unique genes that have been lost during domestication. The detection of stress-tolerance genes can be applied to produce salt-tolerance crops through de novo domestication using CRISPR/Cas9 system. On the other hand, halophytes provide an alternate strategy to cope with the salt stress and can be exploited in breeding programs to develop new crops with increased salt tolerance or improve the current cultivated crops.

Hence, the first prerequisite for *de-novo* domestication is to identify the stress-tolerant genes from CWRs by using high-throughput genomics and transcriptomics approaches. Advances in NGS technology provide a robust, accurate, and economical route to sequence the whole genome of any species. The discovery of numerous domesticated genes along with the emergence of trending techniques for genome editing collectively offer an exciting roadmap leading to the development of climate-resilience crops *via de-novo* domestication. A general workflow for developing salt tolerance crops through *de novo* domestication is presented in Figure 3.

## Transcriptome Profiling of Crop Wild Relatives

The CWRs generally have diversified genomic pool and greater genetic variations as compared to the domesticated species and, thus, give breeders a diverse range of genetic resources including the genes for different stress tolerance (Luo et al., 2017). Transcriptome profiling is the most extensively used technique worldwide to analyze and identify the genetic regulators, responsive genes, or novel alleles under stress conditions (Kashyap et al., 2020). It is quite helpful to annotate the new genomes and assist to study the crop species whose genomes have yet to be sequenced (Long et al., 2019). NGS has eased the designing of RNA-Seq technologies and *de-novo* transcriptome assembly (Kashyap et al., 2020) that permit the identification of unique transcripts and gene expression variations under salt stress in CWRs and other important crops. Also, these sequencing techniques have crucial applications for genome-wide screening of differentially expressed genes (DEGs) and transcription factors (TFs) responsible for salinity tolerance (Wu et al., 2017).

Transcriptome profiling can provide variations in allelic expression of genes portraying the orthologues and paralogues in the study of polyploidy genomes that can be crucial to understand the genomic roots of stress tolerance in CWRs, landraces, and domesticated crops (Brozynska et al., 2016). Transcriptome analysis of CWRs using *de-novo* assembly under varying salt concentrations reveals the difference in expression profile of various differential expressed unigenes, related TFs, and candidate genes that provide salinity tolerance to plants (Kashyap et al., 2020).

There are numerous studies that have been conducted to investigate the salt tolerant mechanism by sequencing transcriptome of CWRs and identified numerous stress-responsive genes and TFs that can be used for *de-novo* domestication. For example, transcriptome sequencing of a wild relative of halophytic potato species (*Ipomoea. imperati*) was executed to identify the high-salt tolerant genes. *Ipomoea imperati* is a diploid halophytic wild relative of cultivated potato and harbors rich sources of genetic resources including high salinity tolerance. Comparative analysis of transcriptome profiles under normal and salt stress conditions was performed to elucidate the stress-responsive pathways and corresponding candidate genes. *De-novo* assembly generated 67,911 total transcripts with 39,902 putative genes annotated from which 220 and 936 salt-responsive genes were found in leaves and roots, respectively. Many candidate genes were identified that have been involved in several stress signaling pathways like transporters, ABA signaling, metabolic enzyme synthesis, TFs activities, and antioxidant enzyme pathways. Two transporter genes were also detected including inositol transporter and vacuole cation/proton exchanger that was supposed to reduce the harmful effects of salt stress (Luo et al., 2017).

*De-novo* transcriptome analysis was carried out to elucidate the salinity tolerant genes in wild tomato (*Solanum chilense*) which has excellent ability to endure extreme salinization. Comparative gene expression of wild and cultivated lines was studied by using RNA-sequencing and obtained 386 million clean reads. Further, *de novo* assembly revealed 514,747 unigenes from which 265,158 were differentially expressed. A total of 134,566 DEGs were up-regulated under salinity stress which involved in various signaling pathways including gibberellin, abscisic acid, auxin, ethylene, and cytokinin. In addition, novel tolerant genes encoding TFs, homeostasis, stress response, osmotic regulation, transporters, ROS scavenging system, arginine, and proline metabolism were accumulated in wild line as compared to cultivated tomato (Kashyap et al., 2020). *De-novo* transcriptome analysis of *Gossypium aridum* was performed to assemble 98,989 unigenes from 41.5 million retrieved transcripts. Various DEGs were up- and down-regulated under varied concentration of salinity stress and involved in stress signaling, transport, and hormone stimulus pathways. The genes controlling the transporter activity and protein kinase activity were significantly up-regulated and provide salt tolerance to *Gossypium aridum* (Xu et al., 2013). In another report, Wei et al. (2017) investigated the salinity tolerance of wild cotton (*Gossypium klotzschianum*) by investigating the gene expression patterns and hormonal fluctuations subjected to salt stress through transcriptomic profiling of roots and leaves. RNA-seq analysis retrieved 37,278 unigenes and identified ~14,000 differentially expressed genes in both roots and leaves that are supposed to be involved in salinity tolerance. Gene functional analysis elucidated the role of some genes in ion homeostasis, signal transduction, and salt overly sensitive mechanism under salt stress. This data allowed an understanding of the broaden mechanism of salinity tolerance in cotton and provided rich genetic resources for cotton genetic improvement.

Identification of key regulatory networks is crucial to understand the salinity tolerance mechanism as very little information is available for wild relatives of major crop species. Wu et al. (2017) carried out *de novo* global transcriptome profiling of tartary buckwheat (*Fagopyrum tataricum*) to identify potential salt-tolerant regulators. The results indicated 57,921 unigenes from 53.15 million clean reads which also included 544 DEGs and several salt responsive genes encoding for abiotic-related TFs, ATP-binding cassette (ABC) transporters, heat shock proteins, phosphatase, and protein kinases (Wu et al., 2017).

Investigating the key molecular and physiological networks in response to salinity is vital for the development of salt-tolerant crops. Wild relatives of sweet potato such as *Ipomoea pes-caprae* harbor unique genes for salt tolerance that could be utilized in different breeding programs to develop new crop varieties with improved salinity tolerance. Liu et al. (2020b) conducted RNA-seq analysis of *Ipomoea pes-caprae* to mine regulatory networks controlling different salt tolerance mechanism. The results showed 40,525 genes from which 3,334 genes were differentially expressed in leaves while 2,478 genes were differentially expressed in roots in response to salt stress. Several candidate genes functioning in abscisic acid (ABA) signal pathway, hormone signal transduction, and the mitogen-activated protein kinase (MAPK) signaling pathway were also identified under salt stress. Zhou et al. (2016) performed transcriptome analysis of *Oryza rufipogon*, a wild progenitor of cultivated rice, to study the genetic mechanism of salt tolerance. The results identified many transcripts (6,867) differentially expressing in different tissue from which 3,105 were up-regulated in roots and 2,216 up-regulated in leaves. Several salt-responsive genes have been discovered that were co-localized on salinity tolerance associated loci identifying potential candidate genes for salinity tolerance in rice. RNA-Seq of wild barley (*Hordeum spontaneum*) leaves was conducted subjected to salinity stress and yielded 115 million reads. The results showed increased expression level of DEGs which regulate numerous biological activities such as protein refolding, signaling network, ethylene production, reactive oxygen species (ROS) scavenging, flavonoid biosynthesis, exchanger mechanisms, and electron transport in response to salt stress (Bahieldin et al., 2015).

## Pan-Genomes: Snapshot Genetic Architecture of CWRs and Capture Unique Genes for Stress Resilience

Genetic variations among the genomic pool of new crop cultivars are as a result of continuous modifications extending from SNPs, *via* small indels, to large structural variants (SVs) and can produce significant variations in the gene content (Tao et al., 2019). Access to the reference genomes of major crops species has permitted discovery of the genome-wide association among different traits and establish a connection between genotype and phenotype. But resequencing techniques based on single reference genomes have been the main constraint for complete understanding of genetic variations and cannot detect the SVs (Brozynska et al., 2016). A single reference genome does not demonstrate the allelic diversity and is unable to catch the complete genetic diversity because of the presence of SVs (Bayer et al., 2020; Danilevicz et al., 2020). The SVs which also include presence/absence variants (PAVs) and copy number variants (CNVs) limit the capturing of genetic variants and change the genetic information within a species. The CNVs are present in different copy number in all individuals of a species while the PAVs are referred to the extreme form of CNVs as it is either present in some individuals or completely missing in others (Hirsch et al., 2014).

Recently, the concept of pangenome has revolutionized the genomic research, and a huge wave of crop pangenomes have been reported in the last 6 years. The assembly of crop pangenomes is essential to calculate the range of genetic variations in order to obtain the full landscape of a gene pool. It has now been extensively used to explore the genomic composition and variation among individuals of a species (Bayer et al., 2020; Danilevicz et al., 2020). Crop pangenomes allow multidimensional analysis of polymorphism and SVs in genomes and compare various genomes to investigate the genomic variation among individuals of a species or higher taxonomic groups simultaneously. It can also pave the way for understanding the evolutionary process and optimizing precision breeding for crop improvement (Zhao et al., 2018). This evolving idea harnessed with the third-generation sequencing provides an excellent platform to recapture eliminated genes, unravel new genes, and expand our knowledge to decipher the genome dynamics and organization.

Pangenomes have two major categories like open pangenomes and closed pangenomes (Tettelin et al., 2005). It refers to the complete repertoire of genes present in a group of individuals belong to the same species and composed of two parts: core genome that includes all the core sequencing/genes present in all individuals whereas variable (accessory or dispensable) genomes consist of variable genes that are shared by only some individuals (Bayer et al., 2020). The dispensable genes can further be segmented into unique genes that are only found in a single individual and some genes shared by two or more members of a species. Recent studies have disclosed that the core genome is generally larger than the variable genome and covered the major share of pangenome. To the viewpoint of evolution, the core genes remain conserved and are very important to accomplish the crucial functions within a species (Danilevicz et al., 2020).

On the other hand, dispensable genome contains the genes that provide genetic diversity to the species which assist the individuals to survive under harsh climatic conditions and contribute many agronomic characters. Therefore, variable genes are reported to be evolving quicker than core genes. The primary crop pangenomes assemblies revealed the presence of large variable genes of about 15–40% in each species and the genes that show PAV are normally described with anticipated functions linked with several abiotic and biotic stress tolerance (Bayer et al., 2020). Pangenome analysis of dispensable genome also supports the comparison between exotic and wild cultivars which helps to examine the gene content in wild species that have been lost during crop domestication. This strategy can capture unique genes that are missing in reference genome and may offer greater stress resilience to climate change (Zhao et al., 2018). In addition, accessory genes are regulated by SV and are considered as the main factor for phenotypic variations of yield-related traits. So, pangenome analysis permits a comprehensive image of complete gene content of a species and offers inclusive knowledge of how selection during domestication may modify the rate of dispensable genes (Tao et al., 2019).

Until now, pangenomes of some important crops have been annotated and mapped including rice (Schatz et al., 2014), legume (Hu et al., 2020), wheat (Montenegro et al., 2017), soybean (Liu et al., 2020c), maize (Hirsch et al., 2014), tomato (Gao et al., 2019), and sunflower (Hübner et al., 2019). These discoveries led the plant domestication into a new era as the pangenomes of agronomically vital crops are available and the genes from the CWRs can be reintroduced into any crop by using modern genome editing techniques to develop elite crop cultivars. Researchers are still striving hard to mine the specific stress-related (salinity, drought, etc.) superior domesticated genes from wild relatives through forward genetics or reverse genetics for functional analysis to tackle stressful environmental conditions. At the moment no such study of pangenome has been reported to identify and map the salinity tolerance genes in major crop species. There is the prime need to construct the pangenomes particularly for specific abiotic stresses like salinity to produce the datasets for novel salt tolerance genes which can be used to develop salt tolerance crops in future.

## Crop Wild Relatives Harboring Genes for Salt-Tolerance

Salinity stress is polygenic in nature, linked with several traits, and governed by the multiple genes. The complex nature of salt stress is the major challenge to target all the genes simultaneously during modern breeding platforms for crop improvement. Additionally, intensive selection and breeding to achieve the elite crop cultivars during domestication resulted in the reduction of genetic diversity and loss of important stress-tolerant genes. Therefore, present-day crops are unable to cope with the extremes of climate stresses due to the limited genetic resources (Henry, 2014; Warschefsky and von Wettberg, 2019). For example, it is estimated that 2–4% (1200) of genes have been lost in modern cultivars of maize (*Zea mays*) during its domestication from the maize wild relative (*Zea mays* ssp. *parviglumis*) (Wright et al., 2005). Haudry et al. (2007) reported 70% reduction in the genetic diversity of wheat compared to its wild counterpart wild emmer as a result of domestication, which most probably contained alleles for climate resilience (Huang et al., 2016). Likewise, cultivated soybean and rice also faced a severe 50% reduction in the process of selecting superior traits (Hyten et al., 2006; Xu et al., 2012; Zhou et al., 2015). To cope with this problem, quick efforts need to be taken to study the CWRs to increase the genetic diversity of crops (Solis et al., 2020). This needs to tap the underutilized and unexploited treasure troves of CWRs which can be a potential source of tolerant genes (Dempewolf et al., 2017). The CWRs have the ability to adapt in diverse environmental conditions offering high genetic variations and allowing them to expand their genetic diversity during natural selection. This makes CWRs highly tolerant to different abiotic stresses as compared to modern crops (Nevo and Chen, 2010). The CWRs contain valuable novel genes for different abiotic stresses that can be used to broaden the genetic diversity of cultivated species using next-generation breeding tools (Brunazzi et al., 2018; Tian et al., 2021). The preservation of enriched resources of CWRs has been an utmost goal of breeders to achieve sustainable crop production (Zhang and Batley, 2020). Retrieving the stress-responsive genes that have been lost during the domestication process is crucial for crop improvement. Identification and introduction of these genes from wild to domesticated crops can be vital for climate resilience (Mammadov et al., 2018; Razzaq et al., 2021b).

During the last decades, tremendous progress has been achieved to probe and exploit the rich potential of CWRs to achieve tolerance against abiotic stresses through breeding programs. The CWRs provide a valuable source of salt-tolerant genes that can be introduced into the cultivated crops to develop new crop cultivars having greater salinity stress-tolerance (Mickelbart et al., 2015; Dempewolf et al., 2017). For example, Ganie et al. (2014) studied a diverse collection of rice salt tolerant germplasm including cultivated rice and wild rice (*Porteresia coarctata*). Molecular markers were used to screen the superior lines for salinity tolerance and identified nine quantitative trait loci associated with unique gene *salt*. In another study, wild relative of rice was analyzed under salt stress and detected some unique genes including HKT1;5, *HKT8, HAK6*, and *SKC1* (Quan et al., 2018). Similarly, wild rice (*Oryza rufipogon*), the progenitor of Asian rice, has been studied by three independent groups and reported wild genes for salinity tolerance such as *OrWRKY* (Nan et al., 2020). A gene TmHKT1;5-A from wild relative of wheat (*Triticum monococcum*) showed increased tolerance against salinity as compared to the wheat lines with this unique locus (Munns et al., 2012). Cao et al. (2017) investigated a key NAC transcription factor (*GsNAC019*) from *Glycine soja* in response to salt stress. Overexpression analysis revealed that *GsNAC019* was highly associated with ABA signal transduction mechanism and induces salinity tolerance by controlling the downstream tolerant-genes. In another study, a novel transcription factor of *Glycine soja GsERF71* was analyzed in relation to alkaline stress. The data demonstrated that *GsERF71* promote salinity tolerance by increasing the expression level of H+-ATPase and control the auxin concentration in plants (Yu et al., 2017). Wild soybean contained a proline rich-type gene (GsEARLI17) involved in cuticle formation, higher leaf opening, and improved germination rate when subjected to salinization. Also, lower malondialdehyde concentration increases chlorophyll content and higher salinity tolerance exhibited by GsEARLI17 gene (Liu et al., 2015).

Wild relatives of other important crops also provide the salinity tolerant genes including *SOS* and *RAB* in wild sugarcane (Kasirajan et al., 2020), *ZmHKT2* in wild maize (Cao et al., 2019), and *HAI1, HAB2* (Liu et al., 2020b), *IpDHN* (Zhang et al., 2018a), and *IpSR* (Zhang et al., 2018b) in wild potato (*Ipomoea pes-caprae*).

## *De-novo* Domestication *via* Genome Editing Can Be a Promising Strategy for Salt Tolerance

As we begin to take the full picture of crop genomes and identify novel candidate genes or specific loci controlling agronomic traits for stress tolerance, yield, and quality enhancement, there is great potential to investigate the functional genomics of recaptured genes to develop new crop genotypes. It offers valuable resources to interrogate the existing traits in a crop and reintroduce the unique traits from wild to accelerate the domestication process (Fernie and Yan, 2019). This can be achieved effectively through cutting-edge genome editing techniques to manipulate desired gene of interest, directly eliminate the harmful alleles, incorporate useful genes without linkage drag, introduce quantitative variation, and to enhance the frequency of recombination (Li et al., 2018). Genome editing technologies have allowed precise and targeted manipulation to be tailored in any plant genome and now revolutionize crop breeding by accelerating crop improvement. Precision breeding *via* gene editing approaches helps to improve the desirable trait of interest with minimum risk-related issues and thus takes the future of agriculture to the next level (Razzaq et al., 2019).

Clustered regularly interspaced short palindromic repeat/CRISPR-associated (CRISPR/Cas9) system has emerged as the most powerful genome editing tool for crop improvement. CRISPR/Cas system is continuously evolving by introducing the new Cas variants such as Cas9, Cas10, Cas12, and Cas13 orthologs into its toolbox and overcome the drawback of zinc-finger nucleases (ZFNs) and transcriptional activator-like effector nucleases (TALENs) (Puchta, 2017). Also, multiplex genome editing, base editing, DNA-free genome editing, and prime editing have opened new horizons in crop genome editing (Tyagi et al., 2020). These innovations in CRISPR/Cas system improve the editing efficiency by offering novel features like various protospacer adjacent motif (PAM) sites, small protein size, target specificity, low off-target effect, and efficient transformation techniques.

Technological advancement in CRISPR/Cas9 mediated genome editing has enabled development of new crop cultivars through *de novo* domestication by mimicking the natural breeding process (Fernie and Yan, 2019). Some groups have successfully manipulated the domesticated genes in CWRs as a proof-of concept (Li et al., 2018; Zsögön et al., 2018; Yu et al., 2021). Until now, *de novo* domestication has been attained in Solanaceae species and tetraploid wild rice through genome editing technologies (Li et al., 2018; Zsögön et al., 2018; Yu et al., 2021). These studies can be considered as an excellent model system for devising, testing, and executing this techniques for other species.

The successful early reports propose that *de novo* domestication could be a promising strategy for breeding improved cultivars with beneficial traits and boosted/enhanced resilience (Zsögön et al., 2018). For example, Li group performed the *de novo* domestication of four stress-tolerant wild tomato (*Solanum pimpinellifolium*) accessions to accelerate the domestication process through CRISPR/Cas9 mediated multiplex genome editing. The upstream open reading frames, cis-regulatory regions, coding sequences, and genes regulating the ascorbic acid synthesis, fruit production, flower formation, and crop morphology were targeted for editing. The results showed that the edited plant progeny was free of Cas9 and retained the parental domesticated phenotypic traits. The edited tomato accessions displayed improved disease resistance and salinity tolerance features (Li et al., 2018). These findings provide an excellent platform to domesticate wild Solanaceae species and can allow recapitulated breeding programs without affecting the agronomical important traits. Recently, a *de novo* multi-replicon toolbox was designed to target the *SlHKT1;2* allele to produce replicon-free salt tolerance tomato. CRISPR/LbCpf1 system was used to get the stable HDR alleles without incorporation of any selectable marker or antibiotic-resistant gene. Integration of *HKT1;2* HDR allele inherited by all the offspring of self-pollinated plants and confer tolerance to salt stress (Vu et al., 2020).

In another experiment, Zsögön et al. designed multiplex genome editing strategy using CRISRP/Cas9 toolkit to introduce beneficial agronomic traits in wild tomato (*S. pimpinellifolium*) cultivars. *De novo* domestication of wild tomato was achieved by disrupting the six gene loci *LYCOPENE BETA CYCLASE, FRUIT WEIGHT 2.2, OVATE*, and *SELF-PRUNING* that resulted in the improvement of fruit size, yield, and nutritional value of the tomato. The morphology of edited plants was changed along with the nutritive composition of fruit. The edited lines produced ten-fold more fruits with three-fold larger fruit size and 500% increased accumulation of lycopene as compared to the wild parent. This experiment led to the foundation for *de novo* domestication to accelerate the molecular breeding and exploit the genetic diversity of CWRs for crop improvement (Zsögön et al., 2018). Allotetraploid wild rice has greater genetic diversity and contains genes for higher biomass production and stress tolerance as compared to the cultivated rice. However, allotetraploid wild rice cannot be cultivated as a staple crop because of some undesirable features such as poor grain quality, low grain yield, easy seed shattering, long awn, sparse panicle, tall architecture, high photoperiod sensitivity, and sprawling growth habit (Xie and Liu, 2021). Recently, *de-novo* domestication of allotetraploid wild rice (*Oryza alta*) was performed to target the six potential agronomically important genes including abiotic stress, biotic stress, heading date, sterility, nutrition use, yield, and quality (Yu et al., 2021). The results revealed that by developing the efficient transformation methods for allotetraploid wild rice, six agronomically important traits could be domesticated successfully in O. alta and improved the undesirable characters. *De-novo* domestication of wild rice provides the evidence that the allotetraploid can be transformed into a new cereal crop. It might be a breakthrough event to improve the wild relatives of the cereal family and can be extended to other member of species for developing climate resilient crops.

As salinity tolerant is a multi-genic trait that is controlled by different genes, and practically it is impossible to acquire the salinity tolerance only by targeting a single gene. It may require targeting the group of genes simultaneously that control the salinity tolerance mechanism. Therefore, *de-novo* domestication can be a promising strategy to target the multiple genes at the same time to develop salt tolerance in crops.

## Halophytes: An Alternative Route Towards Salt Tolerance

In addition to advancement in molecular technologies, domestication of halophytes is another efficient way forward to acquire salinity tolerance in crops. Halophytes are inhabitants of extreme saline environments which makes them extremely tolerant to salt stress. The fascinating physiology of halophytes provides them excellent adaptability to thrive in harsh environments where most of the glycophytic plants are unable to live (Flowers and Colmer, 2015). Halophytes are not only tremendous model plants to study the salt tolerance mechanisms but also have promising potential to cultivate as crops for food, fiber, animal feed, and industrial purposes in salt-affected lands (Rozema et al., 2013; Panta et al., 2014). The exploitation of halophytes for salinity tolerance needs a deep insight snapshot that the evolution of major grain crops, glycophytes, and halophytes has achieved as a result of completely different processes (Cheeseman, 2015). Therefore, it is vital to study the various tolerance networks functioning at different levels, ecological, physiological, molecular, and biochemical, for a better understanding of the halophytes (Rozema et al., 2015). The long-term objective of these investigations is to harness the genetic resources of halophytes for improving salt-tolerant traits of crops in salt-affected soils and re-vegetations of saline soils (Flowers and Colmer, 2015). Also, halophytes are supposed to be a valuable source for tolerant genes which can be *de-novo* domesticated to develop new salinity tolerant crops. Ventura et al. (2015) developed a database for halophytes named eHALOPH that can be accessed from (http://www.sussex.ac.uk/affiliates/halophytes/) to gather all the information about halophytes at one place.

Many species of halophytes have the great potential to be utilized as food crops and can contribute to future agriculture to cope with the soil salinization problems. Some of the halophytes have greater genetic resources but are under-utilized due to less available information and research (Panta et al., 2014). There is a prime need to explore the untapped resources of halophytes. For example, quinoa (*Chenopodium quinoa*) is an important halophytic species largely grown in many countries, due to its ability to endure multiple stresses and offer better nutrition as compared to major cereals. Quinoa is considered as a future crop to meet the increasing food demands of people and lessen the pressure on the world's agriculture system to ensure global food security (Ruiz et al., 2014; Zou et al., 2017). Panuccio et al. (2014) demonstrated the excellent potential of quinoa to grow in highly saline soils or the presence of seawater due to activation of certain metabolic and efficient antioxidant mechanisms triggered by salt stress which help the quinoa plants to adjust osmotic homeostasis at different growth stages. Recently, a well-annotated draft genome of halophytic quinoa was assembled that contains about 54,438 protein-coding genes from which 99% belong to glycophylic orthologous genes. The genome sequencing of quinoa gave valuable information about the evolution of halophytes, and provided a basis for molecular breeding by identifying unique genes associated with salinity tolerance and nutritional composition (Zou et al., 2017). Sun et al. (2017) investigated the comparative response of glycophyte pea (*Pisum sativum*) and halophyte quinoa under salinity stress. The results indicated the higher salt tolerance by quinoa due to the rapid elimination of cytosolic Na^+^ and hyperaccumulation of K^+^ in different tissues as compared to glycophytic pea.

Sea beet is another important halophytic species that can serve as a good source for salinity tolerance genes and can be domesticated with cultivated edible beets such as sugar beet, red beet, table beet, and fodder beet to develop salt-tolerant varieties. These beet varieties are supposed to be derive from their ancestor sea beet through the domestication process over the period of many 100 years. Rozema et al. (2015) compared the salinity tolerance level of sea beet (*Beta maritima*) as compared to the cultivated beet lines in hydroponic conditions to investigate whether the tolerance level has been increased in cultivated beet by recent breeding approaches. The relative growth rate and some other important agronomic traits leading to high sugar contents in cultivated beet were calculated and found a slight decrease in salinity tolerance as compared to sea beat.

*Suaeda salsa* is regarded as a promising halophyte that can be utilized as forage, food, and medicine like quinoa and beet. *Suaeda salsa* not only provides a beneficial model to explore the salinity tolerance mechanism but also acts as a rich source of genes that may regulate the complex salinity tolerance mechanism. To date, several genes linked to ion homeostasis have been cloned, and these can be used in different breeding programs (Song and Wang, 2015). Li and Song (2019) performed targeted metabolic proofing to analyze the differential metabolites under salinity stress conditions to evaluate the nutritional value and salt tolerance of *S. salsa*. Metabolic annotation revealed the presence of some amino acids, organic acids, antioxidants (quercetin), and lipid (α-linolenic acid) that may be associated with osmotic homeostasis. Boestfleisch et al. (2014) identified *Salicornia europaea, Lepidium latifolium, Plantago coronopus*, and *Tripolium pannonicum* as a potential source of nutraceuticals and functional foods. Different salt treatments control the accumulation of ROS scavenging enzymes, glutathione oxidation/reduction, ascorbate, flavonoids, and phenols in different families of halophytes. Members of the Salicornioideae family have excellent ability to grow in saline lands and can be characterized for future breeding. Singh et al. (2014) collected diverse varieties of halophytic Sacroconia and Salicornia species across UK, Netherlands, Kazakhstan, Israel, and Germany for external transcribed spacer (ETS) sequence analysis which indicated a strong divergence among two genera, but some Salicornia species were less divergent. Under artificial growth conditions, different Salicornia varieties showed high biomass production under salt treatments.

A wheat halophytic relative (*Elytrigia elongata*) also known as tall wheatgrass is utilized to increase the performance of wheat cultivars in salt-affected soils in China. Guo et al. (2015) investigated the salt tolerance mechanism in salt-tolerant decaploid *E. elongate* and salt-susceptible tetraploid *E. elongate*. The salt-tolerant varieties have powerful selective capacity for K^+^ in place of Na^+^ leading to maintain the ion balance and provide high salinity tolerance in decaploid *E. elongate*. Sea barley (*Hordeum marinum*) is highly tolerant to salinization conditions over cultivated barley. Comparative analysis of HvHKT1;5 cultivated barley transporter and HmHKT1;5 sea barley has been performed to characterize homologous transporters under salinity stress. The results suggested that HvHKT1;5 regulate the absorption and translocation of Na^+^ while HmHKT1;5 showed a much weaker ability to uptake Na^+^ and completely lack the function of Na^+^ translocation from roots to shoots (Huang et al., 2019). In an earlier study, Huang et al. (2018) uncovered the molecular differences between sea barley and cultivated barley at transcriptome and metabolome level under salinity stress. The results revealed several ion homeostasis mechanisms and some unique ROS detoxification-related genes in response to salt stress in sea barley. These may be the reason sea barley demonstrate greater salinity tolerance compared to cultivated barely. Ventura et al. (2014) studied a naturally occurring halophyte (*Crithmum maritimum*) that could be utilized as a cash crop in extremely saline soils. Their results exhibit variations in nutritional contents, leaf metabolites, and overall plant growth among different lines originated from different regions under salt stress.

## Conclusion and Outlook

Food security and climate change are the main reasons for continuous innovations in modern breeding approaches in order to produce enough food for a rapidly growing population. Soil salinization is one of the major limiting factors to achieve global food security. The conventional crop domestication is now unable to accelerate crop production due to the loss of genetic diversity during the selection of elite crop traits. The CWRs that harbor the stress-responsive genes are very crucial breeding resources to fast-track the crop improvement programs. *De novo* domestication has emerged as a powerful strategy to accelerate the natural breeding process by reintroducing the unique domesticated genes from the CWRs to cultivated genotypes and also to domesticate the CWRs with agronomically important traits to improve their yield and nutritional value.

Recent progress in genomics and transcriptomics tools has enabled us to understand the genetic mechanism underlying salinity stress. Transcriptome profiling offers an excellent platform to map the key genes from CWRs and other cultivated crops that confer salt tolerance. Innovations in NGS technology allow construction of high-quality pangenomes of several crops that provide a complete picture of genetic diversity among the individual and collect the whole gene pool present in a species including the core and dispensable genes. This provides an unprecedented opportunity for *de novo* domestication by manipulating the stress-responsive genes with greater accuracy through modern genome editing tools like the CRISPR/Cas9 system. So, *de novo* domestication of CWRs and landraces can accelerate the adaptive introgression of unique genes and speed up the crop breeding to develop climate-ready future crops having greater salt resistance and higher yield.

There is still some breakthrough needed in these techniques to overcome certain limitations and pitfall remains. There is a need for next-generation sequencing technologies that can handle the multidimensional genomic information and analyze the whole genetic content without losing any information. In addition, the construction of super-pangenomes provides full access to genetic variations at the genus level and restores a huge number of key genes that can be used to develop climate-smart crops.

*De novo* domestication by using genome editing can produce highly nutritive stress-resilient new crop variety in a short time with greater accuracy. However, limited knowledge about the CWRs and protocol optimization for their transformation are the technical hurdles to achieve this goal. Furthermore, the regulatory affairs and societal and public safety concerns related to genome-edited crops are currently the major hurdles to approve the gene edited crops globally. Due to strict regulatory rules and risk assessment protocols, all type of gene-edited crop research is banned in all European countries. This makes CRISPR/Cas based genome edited technology imperfect to apply in breeding programs for crop improvement. Therefore, *de-novo* domestication is not the only solution to develop salinity tolerant crops and needs to rely on other breeding resources to fulfill the food demands of a growing population.

Halophytes-led breeding strategy is another useful strategy to develop new crop varieties by harnessing the potential of highly salt tolerant ancestors of crop species. There are several halophytes species that can be utilized in breeding programs to improve the crop varieties through traditional or molecular approaches. However, limited information about these highly salt-tolerant species is a big hurdle to using them on a large scale.

With the newly developed techniques it can be possible to study the halophytes for salt tolerance and also try to find new halophyte relatives to broaden the genetic stocks for salinity tolerance. In the future, engineering the salt bladders and trichomes in crop species using highly sophisticated genome editing technologies can open promising horizons for improving salt tolerance in crops. Furthermore, the advent of next-generation genome editing tools like prime editing and base editing has the ability to replace a single nucleotide with greater precision and does not involve any insertion or deletion of a gene in coming years. This can help to create targeted mutations in the crop genomes without insertion of any foreign DNA. This will lead to by-pass of the regulatory affairs and minimize the concerns related to public safety and health. In the future, these different *de novo* domestication strategies can revolutionize global agriculture by overcoming all the current drawbacks and fulfill the task of global food security.

## Author Contributions

AR conceived the idea and wrote the first draft. AR and SW initialed the conception and structure of this review. FS helped in initial draft preparation. SW reviewed, edited, and finalized the manuscript. SA, HA, AA, FA, NT, and HE contributed substantially in the ideas, structure, and writing editing of the manuscript during review. All authors listed have made substantial, direct and intellectual contributions to the work, and approved the manuscript.

## Funding

This research was funded by the Deanship of Scientific Research at Princess Nourah bint Abdulrahman University through the Fast-track Research Funding Program to support publication in the top journal (Grant No. 42-FTTJ-76).

## Conflict of Interest

The authors declare that the research was conducted in the absence of any commercial or financial relationships that could be construed as a potential conflict of interest.

## Publisher's Note

All claims expressed in this article are solely those of the authors and do not necessarily represent those of their affiliated organizations, or those of the publisher, the editors and the reviewers. Any product that may be evaluated in this article, or claim that may be made by its manufacturer, is not guaranteed or endorsed by the publisher.
